# 

**Published:** 1887-09-03

**Authors:** 


### The Hospital.

**An Institution, Family, and Parochial Journal of Hospitals, Asylums, and all Agencies for the Care of the Sick, Criticism and News.**

SATURDAY, SEPTEMBER 3RD, 1887.

### Notice to Advertisers.

The scale of charges for small prepaid Advertisements—Situations Wanted, Situations Vacant, etc., etc., is as follows :—

| | s. | d. |
|---|---|---|
| One insertion of three lines | 1 | 0 |
| Three „ „ „ | 2 | 6 |
| Per line afterwards | 0 | 6 |

*A line averages eight words.*

All copy for Advertisements must be sent to A. P. Watt, 2, Paternoster Square, E.C., by first post on the Tuesday *preceding* publication.

### NOTICE TO CORRESPONDENTS.

All correspondence must be written on one side of the paper only.

We cannot undertake to return MSS. not used.

**Communications,** whether intended for insertion or for private information, must be authenticated by the names and addresses of the writers, not necessarily for publication.

**Local papers** containing reports or news paragraphs **should be marked.**

It is especially requested that early intelligence of **local events of interest,** or which it may be thought desirable to bring under notice, may be sent direct to the Editor.

Communications respecting editorial matters should be addressed to the Editor, Norfolk House, Norfolk Street, Strand, London, W.C.

### The Literary Prize.

A prize of **Two Guineas** will be given every month for the best story or incident based upon hospital, or asylum, or student life, or any incident connected with the professions of medicine or nursing.

### Questions and Answers.

All questions should be addressed to Mr. Howard J. Collins, Secretary, The Hospitals Association, Norfolk House, Norfolk Street, Strand, W.C.

Could anyone kindly recommend a home where a girl of eighteen, subject to occasional fits, could be received, in the hope of getting better, or any place where she could earn her livelihood? Parents are poor.

C. G. C.

M. A. H.—We are not aware of any Home specially set apart for nurses who require rest, though such is much to be desired; but M. A. H. will probably find that the house belonging to the St. John's Sisterhood at 34, Mortimer Street, W., or the Home of Rest, Sea View Square, Herne Bay, would meet her requirements.—[Ed. T. H.]

### The Children's Corner.

**Fourth Quarterly Prize Competition.**
Began 2nd July, 1887 ; ends 24th Sept., 1887.

**Puzzles—Tenth Week.**

No. 38. English Towns Anagrammatically Expressed. 1. A tree and part of a pig. 2. A fruit and a preposition. 3. A river and part of the nose. 4. A French cook, and open country.

```
      *
    . * .
  . . * . .
* * * * * * *
  . . * . .
    . * .
      *
```

No. 39. Diamond Puzzle. 1. A consonant. 2. A Spanish title. 3. A fruit. 4. My whole. 5. A trick. 6. A vehicle. 7. An English river.

No. 40. Charade.
My *whole* is of my *second* made,
And by it is my *first* convey'd.

No. 41. A gentleman had an estate, consisting of a square portion of land. He left to his eldest son one-fourth square portion, directing that the remaining three-fourths should be divided among his four youngest sons in similar and equal portions. How was the land marked out ?

**Letters—Tenth Week.**

Next week tell me something about your favourite " Outdoor Games."

**Results of Eighth Week—Puzzles.**

No. 30. Biographical Acrostic.
V an Trom P
A lhambr A
N apol I
D ryde N
Y ach T
C atherin E
K nelle R

No. 31. Co-nun-drum = Conundrum.

No. 32. Buried Islands. Arran, Candia, Achil.

No. 33. Riddle-me-ree. I have had several clever answers to this. Skye and Ossian suggest: " Because while the Queen reigns, her sailors must sail under a Victorious (Victoria's) flag." A. C. K. says: " Because as long as she lives they will have ' Queen's weather' "—fair or fine weather ? but the correct answer is, " Because whilst the Queen lives there will be no Rex" (wrecks).

---

*All right*—Umslopogaas ; *Three right*—A. C. K., Scrooge, Toby, The Eda, Agatha ; *Two right*—A. G. S. McCulloch, Poodle, Ossian, Skye ; *One right*—Princess Ida, Socrates.

**Letters.**

The best letter on Butterflies is from Toby, which, notwithstanding that last week I inserted a letter of his, is so good, that I must again let his appear this week for the benefit of those little correspondents who take an interest in the subject, but have few opportunities of studying it themselves.

When I was living at Epsom I collected some very good specimens of butterflies which I caught during half-holiday expeditions to the Downs and Commons. I have been told that chloroform is the best thing to kill butterflies with, but the chemist would not sell such a dangerous poison to a little boy like me. However, he very kindly offered to kill them for me if I brought them to him. I had also heard that by placing a butterfly in a bottle, and corking it up tight with some crushed laurel leaves, the acid in the leaves would kill it. When I found this means was not effective, I took them to the chemist, who very quickly killed them for me. Among those that I caught were a Painted Lady, a Purple Emperor, a Peacock, a Tortoiseshell, and a Sulphur Yellow, besides several Fretillaries and small Blues. In a short letter like this it would be impossible to give you an idea of the number of butterflies we have in England, but I believe that there are nearly 200 perfectly distinct varieties, not to mention moths, which may be counted by the thousand. In order to get perfect specimens, I also went in for rearing them from caterpillars, and found it most interesting to watch them shedding their skins and changing from larvæ to chrysalides, and again from chrysalides to perfectly formed butterflies. Some of the caterpillars are very beautiful, while some are most ugly: as, for instance, among the former is the woolly bear, that quaint little insect which is the larva of the Tiger Moth ; and from some of the most insignificant caterpillars the most beautiful butterflies are developed. Who would think that the ugly little prickly brown caterpillar that we see feeding on the stinging-nettle would turn into the beautifully-coloured Peacock Butterfly ? The Puss Moth Caterpillar is of a beautiful green colour with red markings, changing its colour occasionally. It has two prongs at the end of its tail, containing pink legs or tentacles, with which it fastens itself to the bark of a tree when changing into its chrysalis state. It then covers itself with bits of the bark with which it is provided. In this little hut it remains until the time comes when it eats its way out, a beautiful, fluffy moth, whose appearance gives it its name of Puss.

Socrates as usual sends an interesting and thoughtful letter. He writes :—

If Butterflies had been the subject of letters for next week, I should have been able to tell you more about them, as I expect to catch some while in Wales. The butterfly is often taken as an emblem of ourselves, for as the caterpillar changes from itself into the character of a butterfly, so we change from mortality to immortality. Last week while in the garden I saw a splendid big Peacock Butterfly, but before I had a chance to catch it, it was over the hedge. I remember, while staying in the Isle of Wight, there were no end of little blue and brown butterflies, with white spots on their wings ; while in Wales last year I saw a large white moth with horns and long white hairs on its legs. I caught it, but not then collecting, I let it go again. I also noticed that the butterflies always go in pairs. They are like bees in some respects,—they live on honey. Although their beautiful wings cannot fold so closely as to let them creep into the Foxglove or Lily-bell, they have tongues like little coils of elastic, which they can put into the petal of any flower as they pass by. Some people say that the butterfly only lives a day, but I know for a fact that a caterpillar got into a bottle, and when it emerged into a butterfly, the neck of the bottle being too small to let it get out again, it lived for two weeks without anything to eat.

Skye's idea of butterflies being like flying flowers which beautify the air as flowers do the earth, is a really poetical one. Princess Ida sends a short letter full of information on the subject. Ossian cannot tell us much about butterflies, but his remarks upon beetles and serpents are amusing to read. He says : " I am sorry I can say very little about butterflies, as I have seen very few down this way. If it were serpents and beetles, I might be better able to write an essay on them, as both are rather abundant in these quarters, too much so indeed to be pleasant. The beetles serve one good purpose : they supply a good meal every evening to the ducks and goslings. The snakes serve no purpose of any great observable moment, unless perhaps to alarm everyone who would like to lie down on the heather to take a rest, and enjoy the view and the pleasant sea breeze. As to butterflies, I frequently with great interest notice the various transformations which the butterfly insects go through from the egg to the perfect butterfly, and especially as seen in the silkworm, a few of which I had last summer in Rochester."

Three marks each :—Toby, Skye, Ossian, Socrates, Princess Ida. Two marks :—Etta.

### Amusements with Profit.

**No. 13. Double Acrostic.**

In church or home life both are found,
*This* strong, *that* weak, yet both are sound.

1. A debt which misers hate, yet pay ;
2. Sometimes a fact, yet fancy's play ;
3. A term which means capitulation,
   Accompanied with degradation ;
4. A name of friendship full of love ;
5. A Norman Bishop, hand and glove
   With Norman William, Harold's foe ;
6. Rather than *this*, the arrow's blow
   Brave Harold met, which laid him low ;
7. My last is what King William won,
   What sluggards hate and burglars shun.

**No. 14. Charade.**

Britannia rules my *first* by means of my *second*,
In spite of every caprice of my *whole*.

**Answers.**

No. 9. Double Acrostic.

G lasgo W

E phemer A

R igh T

M ac E

A rthu R

N erve S

Correct answers have been received from Aralc, Esau, Gretta, Mater, Common Sense, Brownie.

**No. 10. Arithmetical Puzzle.**

The party of friends being represented by 1, 2, 3, 4, 5, 6, 7, 8, 9, 10, 11, 12, 13, 14, 15, arranged their parties as follows :—

| | | | | | |
|---|---|---|---|---|---|
| 1st day .. | 1, 6, 11 .. | 2, 7, 12 .. | 3, 8, 13 .. | 4, 9, 14 .. | 5, 10, 15 |
| 2nd day .. | 1, 7, 13 .. | 2, 8, 14 .. | 3, 9, 15 .. | 4, 10, 11 .. | 5, 6, 12 |
| 3rd day .. | 1, 8, 15 .. | 2, 9, 11 .. | 3, 10, 12 .. | 4, 6, 13 .. | 5, 7, 14 |
| 4th day .. | 1, 9, 12 .. | 2, 10, 13 .. | 3, 6, 14 .. | 4, 7, 15 .. | 5, 8, 11 |
| 5th day .. | 1, 10, 14 .. | 2, 6, 15 .. | 3, 7, 11 .. | 4, 8, 12 .. | 5, 9. 13 |
| 6th day .. | 1, 2, 3 .. | 4, 9, 15 .. | 6. 7, 8 .. | 5, 10, 14 .. | 11, 12, 13 |
| 7th day .. | 1, 4, 5 .. | 2, 7, 13 .. | 6, 9, 10 .. | 3, 8, 1 .. | 11, 14, 15 |

The puzzle may also be worked by taking the first fifteen letters of the alphabet to represent the friends, instead of the above figures.

Correct Answers have been received from V. W., Aralc, Esau, Ellice, Perseverance, Gretta, Mater, Condorcet.

**Notices to Correspondents.**

Man of the Sea.—Letters must reach Norfolk House not later than the first post on *Friday* morning.

